# Compassionate use of ruxolitinib in acute and chronic graft versus host disease refractory both to corticosteroids and extracorporeal photopheresis

**DOI:** 10.1186/s40164-017-0092-3

**Published:** 2017-12-02

**Authors:** Mauricio Sarmiento Maldonado, Pablo Ramírez Villanueva, Pablo Bertín Cortes-Monroy, Veronica Jara Arias, Katherine Soto Donoso, Pablo Uribe Gonzalez, Mauricio Ocqueteau Tachini, Jose Antonio Perez-Simón

**Affiliations:** 1 Adult Hematopoietic Stem Cell Transplantation Program, Pontificia Universidad Católica de Chile, Diagonal Paraguay 362, 6th Floor, ZC 832000 Santiago, RM Chile; 2 School of Medicine, Universidad Austral de Chile, Independencia 641, Valdivia, Chile; 3 Department of Hematology, Hospital Universitario Virgen del Rocío, Instituto de Biomedicina de Sevilla (IBIS/CSIC), Manuel Siurot 41013, Seville, Spain

**Keywords:** Ruxolitinib, Graft versus host disease, Corticoid refractoriness, Extracorporeal photopheresis

## Abstract

**Background:**

Ruxolitinib is a potent inhibitor of JAK1/2 with proven efficacy in myelofibrosis. In recent years, research in graft versus host disease (GVHD) has revealed the role of activation of JAK pathways in alloreactive lymphocytes. Some reports have shown significant responses in refractory GVHD patients.

**Cases presentation:**

In this report we present our experience in 8 patients with acute or chronic GVHD with refractoriness to steroids and extracorporeal photopheresis treated with ruxolitinib. Three patients had acute GVHD (1 pulmonary, 2 cutaneous, 1 multi-systemic) and 5 had chronic GVHD (3 cutaneous); 85% obtained an overall response and 50% a complete response with a tolerable toxicity profile.

**Conclusions:**

In our series, Ruxolitinib was very active as a rescue therapy for patients with acute or chronic GVHD refractory to standard treatment.

## Background

Allogeneic transplantation remains the only curative strategy for many patients with hematologic malignancies including leukemias and myelodysplastic syndromes [1, 2]. Graft vs. host disease (GVHD) is one of the most frequent complications with high morbidity and mortality. First-line treatment with corticosteroids allows to obtain a response rate that varies between 50 and 70% [3]; however patients who did not respond have a poor response rate to second line regimens and have a high mortality [4–6]. Extracorporeal photopheresis (EP) has been consolidated in recent years as strategy second line treatment option for corticosteroid-refractory patients, allowing GVHD control in up to 70% of cases with cutaneous involvement with mild immunosuppressive effect and low rates of opportunistic infections [7]. Unfortunately, some patients are still refractory to corticosteroids and photopheresis, becoming a therapeutic challenge since no standard treatment has been identified. Ruxolitinib is a janus kinase (JAK1/2) inhibitor designed for the treatment of myelofibrosis, and in comparison with placebo or best available treatment, reduce spleen size and transfusional requirements [8]. In a murine model ruxolitinib has shown to induce an intense reduction of IL-1β, IL-6, or IFN-γ and TNF and other cytokines implicated in lymphocyte activation, which is a distinctive of GVHD [9]. With this background, ruxolitinib has been used in patients with refractory GVHD with overall responses in acute or chronic GVHD of 85 and 25% of complete remission. In this report we show our experience of ruxolitinib use in 8 patients with severe manifestations of acute and chronic GVHD who did not respond to EP.

## Case presentations

### Case 1

**Fig. 1:**
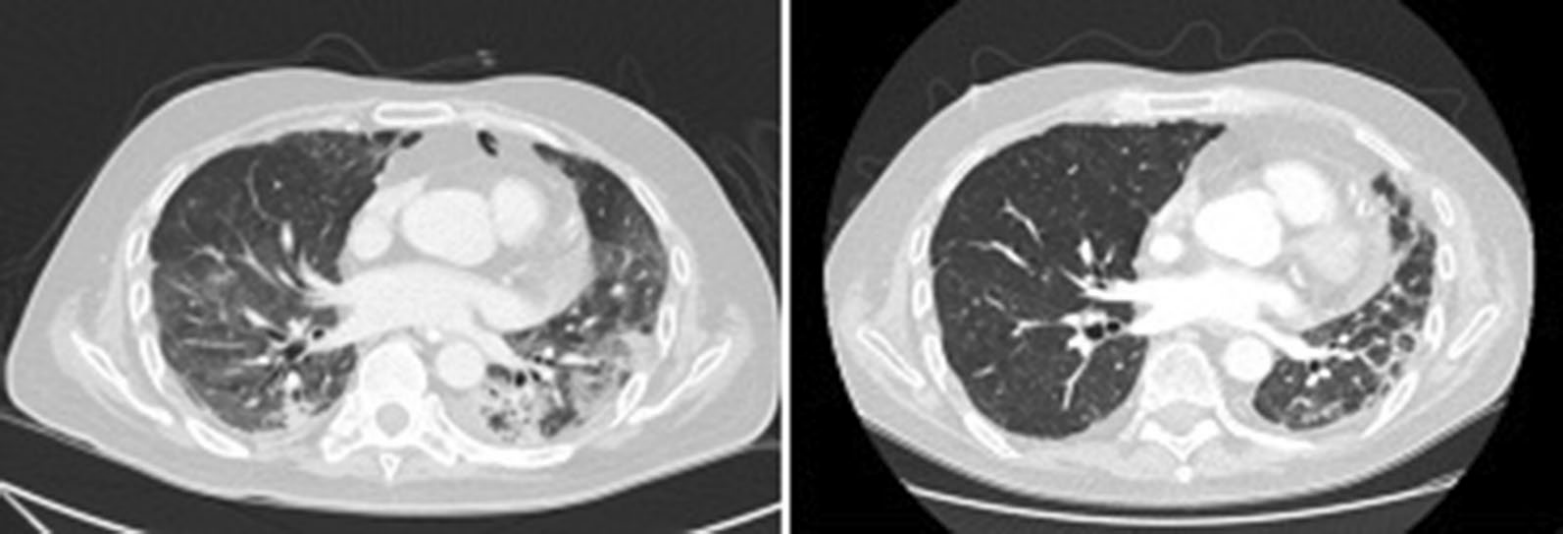
Chest CT scans. Left panel shows an interstitial infiltrate previous to ruxolitinib. Right panel shows 1-month chest CT scan follow-up with nearly complete resolution of the pulmonary infiltrates

A 54-year-old male was diagnosed with Philadelphia (−) acute B lymphoblastic leukemia in 2015. A relapse with central nervous system involvement was demonstrated 4 months after ending Hyper CVAD chemotherapy. He received intrathecal methotrexate with FLAGIDA chemotherapy achieving a second complete remission (CR) followed by a haploidentical myeloablative transplant with cyclophosphamide and total body irradiation (TBI) using peripheral blood hematopoietic stem cell transplantation (PBSCT) from a 3/6 HLA matched sibling. GVHD prophylaxis consisted of post transplant cyclophosphamide (days +3 and +4), tacrolimus and mycophenolate mofetil. 1 week post transplantation he developed febrile neutropenia and a progressive hepatic failure. After engraftment on day 12, he developed a progressive respiratory failure and required invasive mechanical ventilation. At day 14 an evanescent rash was noticed and the skin biopsy showed grade 3 aGVHD. Chest CT Scan showed an interstitial pattern of infiltration without images suggesting fungal infection. BAL was performed an all assays were negative (bacterial cultures, CMV PCR, EBV PCR, aspergillus test, no cardial test). He was started on high doses of methylprednisolone (1.5 g total dose) followed by prednisone 2 mg/kg/day without improvement after 14 days of treatment. A diagnosis of corticoid refractory acute GVHD NIH grade 3 was established and EP was started. During the first session of apheresis, his respiratory function worsened. We started low doses of ruxolitinib (5 mg BID), and after 2 weeks of treatment all cutaneous and hepatic alterations resolved and chest CT scan showed a significant resolution of the interstitial pattern. This allowed the weaning of the 2-month mechanical ventilation. After 12 months of treatment the patient have normal lung function and no other signs of GVHD nor leukemia relapse (Fig. 1).

### Case 2

**Fig. 2:**
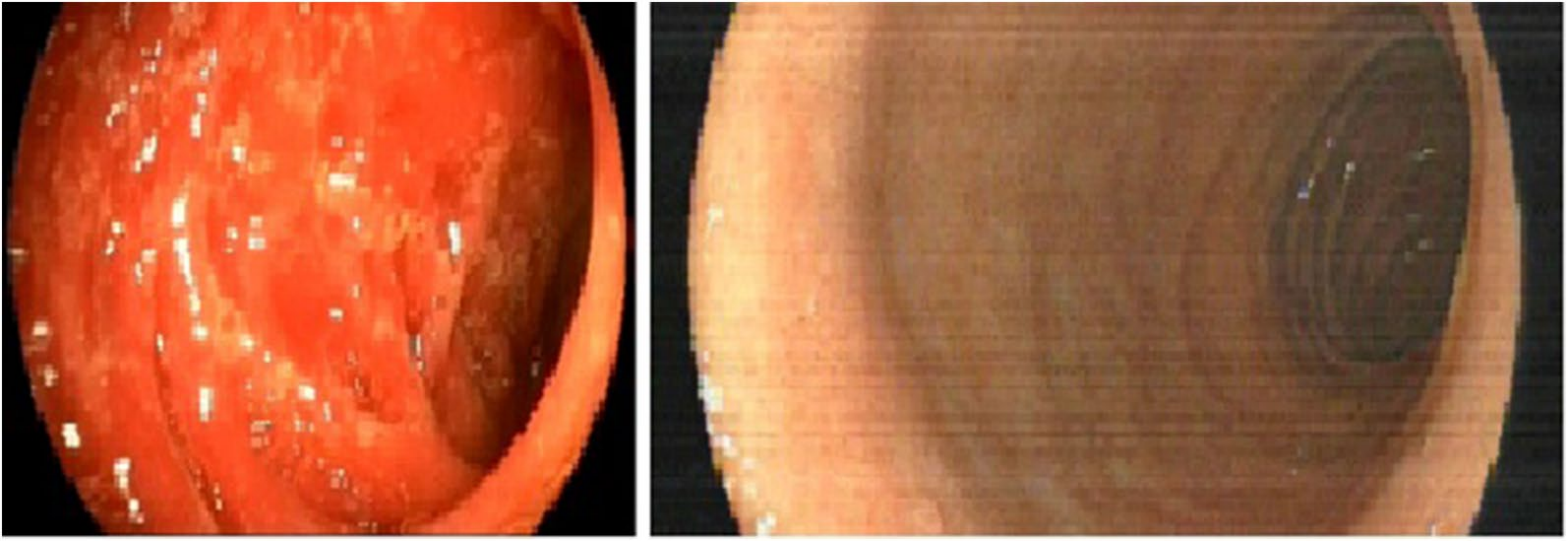
Left panel shows colonoscopy with erythema and inflammation of duodenum. Right panel show a complete resolution of GVHD, 4 weeks after Ruxolitinib

A 28 year old male was diagnosed in 2014 with stage IVB, Hanseclever PS score of 5 classical nodular sclerosis Hodgkin´s disease. He was treated with 3 cycles of ABVD and and interim PET CT showed progressive disease. He then received 2 cycles of ICE (ifosfamide, carboplatine, etoposide) chemotherapy followed by autologous hematopoietic stem cell transplantation (auto-SCT). After 3 month post auto-SCT he was in CR but 6 months later he relapsed. Brentuximab–bendamustine rescue was started and after 4 months of treatment he achieved a second complete remission. Haploidentical transplant with reduced intensity conditioning was performed. At day 45, he developed and intense bloody diarrhea of 2–3 L/day. Endoscopic duodenal biopsy showed a severe GVHD NIH grade 3 (Fig. 2). He received a full dose of corticosteroids during 2 weeks without response, followed by 8 EP sessions, two per week, without clinical improvement. Progressive malnutrition was detected and parenteral nutrition was started. Ruxolitinib was initiated at 10 mg QD, and after 3 weeks of treatment diarrhea was controlled and all of nutritional parameters were improved. After 12 months of treatment with Ruxolitinib, he has normal nutritional parameters, no signs of GVHD and remains in CR.

### Case 3

**Fig. 3:**
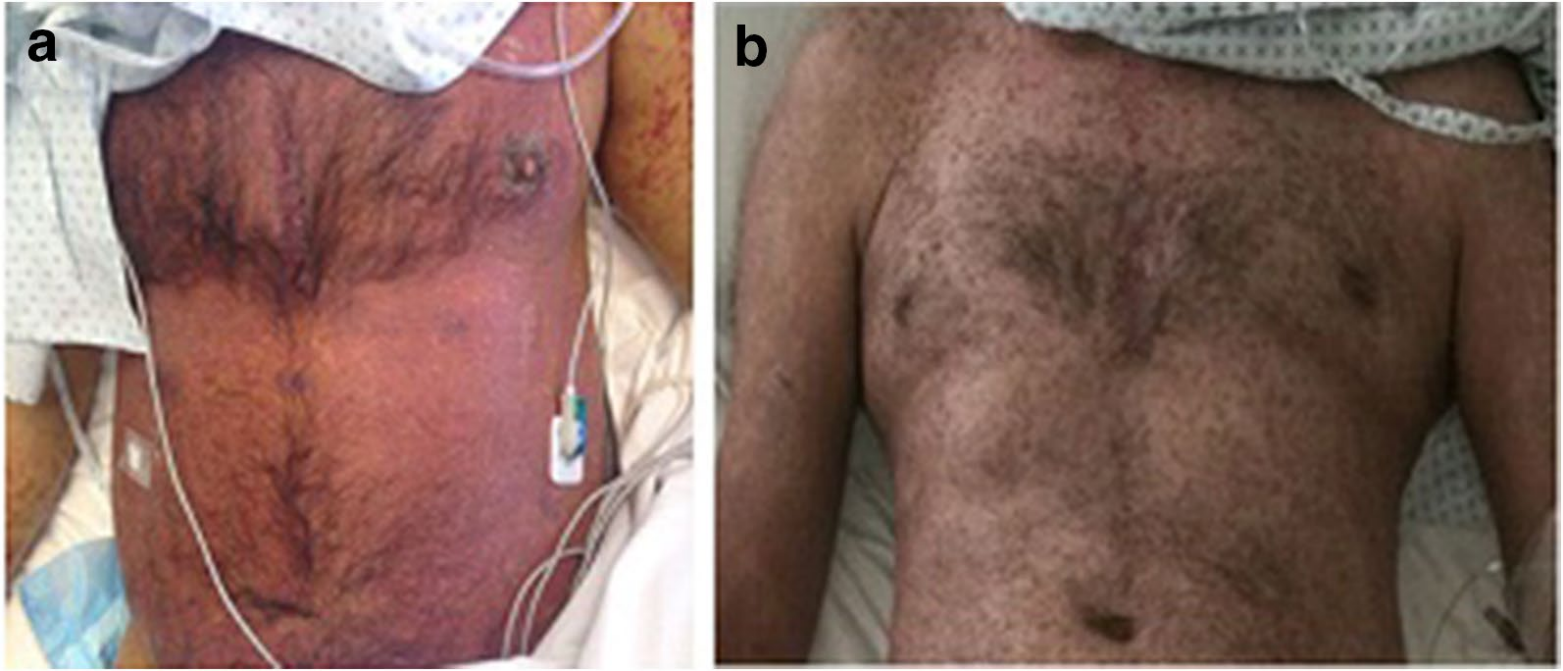
**a** Erythroderma at diagnosis of aGVHD. **b** Regression of cutaneous aGVHD after 2 weeks of treatment with ruxolitinib

A 56-year-old male was diagnosed with germinal mediastinal tumor in 2011 and was treated with 3 cycles of bleomycin etoposide and platinum obtaining CR. A secondary acute myeloid leukemia was diagnosed in 2015. After standard induction with cytarabine and daunorubicin, he underwent a reduced intensity conditioning haploidentical transplantation but a relapse was diagnosed 14 months post transplant. Mixed chimerism with 46% recipient cellularity was found so a first infusion of donor lymphocytes (DLI) at a dose of 1 × 10^5^ CD3/kg was performed. One month later a second DLI of 1 × 10^6^ CD3/kg was performed, which resulted in severe erythroderma with mucosal involvement (Fig. 3). A skin biopsy was performed confirming aGVHD NIH grade 3. 6-Methyl-methylprednisolone was administered without clinical response. After 8 days of treatment he was started on EP twice a week without response. Ruxolitinib was started at a dose of 5 mg BID with significant improvement of his clinical condition. Ruxolitinib is currently being in use 6 months after transplantation. He is in CR and chimerism assay showed 90% donor hematopoiesis.

### Case 4

**Fig. 4:**
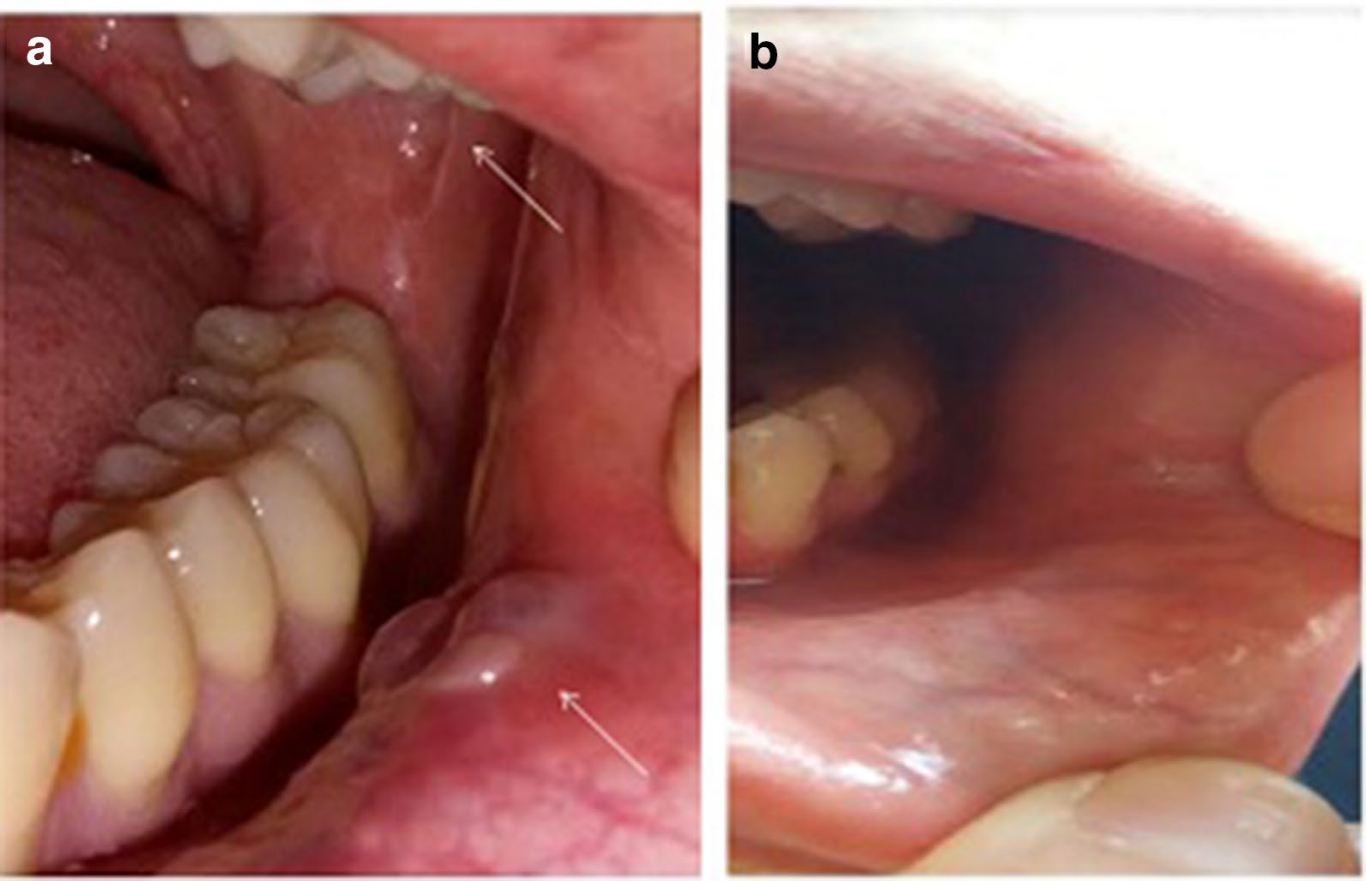
**a** Several mucosal lesions compatibles with mucocele. **b** A nearly complete remission of mucoceles, after treatment with ruxolitinib

A 26-year-old female was diagnosed in 2015 with stage IVB (lung and bone involvement) nodular sclerosis Hodgkin’s lymphoma. She was treated with ABVD chemotherapy. An interim PET/CT scan after 4 cycles showed progressive disease. She received ESHAP chemotherapy with no response after 2 cycles. She received ICE chemotherapy, obtaining CR after 3 cycles. She underwent reduced intensity conditioning haploidentical transplantation in September 2015. 8 months after transplant she developed cGVHD NIH grade 1 with liver involvement (AST 150 μ/L, ALT 190 μ/L, GGT 210 U/L, FA 250 μ/L, normal bilirubin). Other causes of liver injury including hepatotropic viruses, autoimmunity tests, and other parasitic or bacterial infections were ruled out. 6-methylprednisolone 1 mg/kg was initiated. Liver tests remained abnormal. One month later vesicular lesions appeared in the oral mucosa. Biopsy showed mucoceles and inflammation compatible with cGVHD NIH 2. Her liver function progressively worsened and progressive malnutrition was confirmed. She required parenteral nutritional support and ECP was started, but after 8 sessions there was no response. Ruxolitinib 10 mg QD was initiated and after 2 months of treatment the liver tests returned to normal and the oral lesions partially remitted allowing her to satisfactory feeding (Fig. 4).

### Case 5

A 52-year-old male patient was diagnosed with Acute Lymphoblastic Leukemia philadelphia (+) in November of 2012. He was treated with HyperCVAD and dasatinib, obtaining complete molecular remission. He received a 5/6 mismatched unrelated donor transplant after myeloablative conditioning. After 2 years of transplantation he developed a progressive rash, xerophthalmia, xerostomia and oral ulcerations. Besides of cutaneous disease he developed liver malfunction with AST/ALT 4 times above normal values and total bilirubin of 8 mg/dL, concordant with a cGVHD NIH grade 3. 6-Methylprednisolone at 1 mg/kg was started. After 1 month of therapy, liver tests and cutaneous lesions were controlled, but oral lesions worsened. Clobetasol mouthwash was administered without improvement. Malnutrition was evident at this point and ECP was started. After 8 ECP procedures oropharyngeal ulcerations had not improved. We started Ruxolitinib 5 mg BID and after 4 months of therapy a progressive improvement of ulceration was evident. After 2 years of treatment, the patient has mild xerostomia without other signs or symptom of cGVHD.

### Case 6

**Fig. 5:**
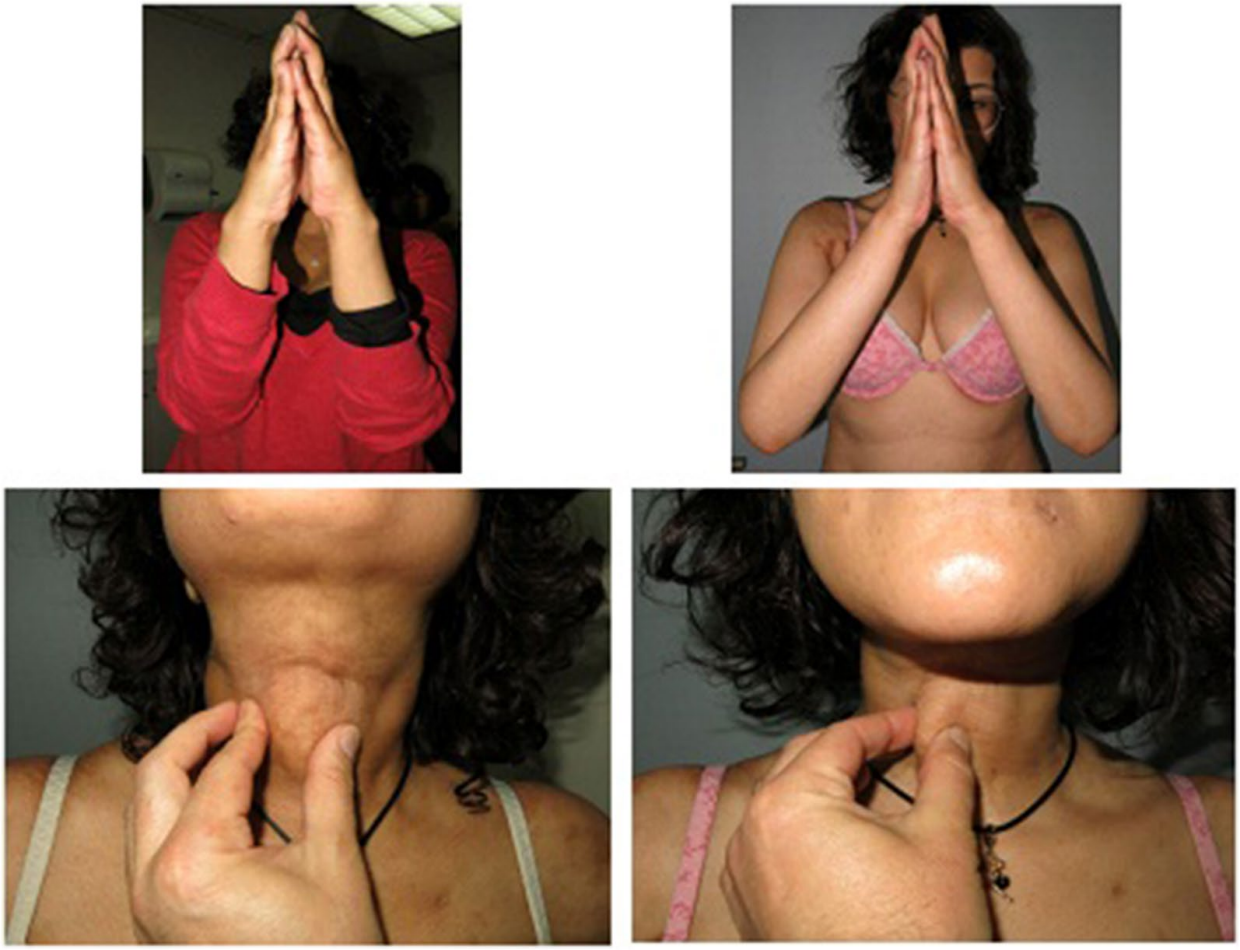
Left panels show intense sclerotic features of cGVHD in hands and neck. Right panel shows some improvement in hands and nearly complete resolution in neck after ruxolitinib treatment

A 36-year-old woman was diagnosed with Ph (+) acute lymphoblastic leukemia in 2013. She was treated with HyperCVAD chemotherapy and dasatinib, with complete molecular remission after 4 months of treatment. She received a PBSCT from her HLA identical brother after a myeloablative conditioning (CY-TBI) and GVHD prophylaxis with methotrexate and cyclosporine. She developed aGVHD NIH grade 1 with intestinal involvement initially treated with budesonide. 24 months later she started with hyperpigmentation in thorax and severe and rapidly progressive sclerodermoid features in the legs, arms and neck, with deep panniculitis and fasciitis-like sclerotic cGVHD NIH grade 3. Skin biopsy confirmed cGVHD, and systemic corticosteroids and low-dose oral methotrexate were started, with only partial response after 2 months of therapy. There was no clinical improvement after 4 months with progression of her scleroderma with severe involvement of movement in the legs and hip rotation. There was no response to ECP 12 sessions. Ruxolitinib 10 mg QD was started and scleroderma improved significantly after 3 months allowing a normal deambulation. After 11 months of ruxolitinib, sclerodermic form persists but in lower grade and normalization of skin changes. Modified Rodnan score improved from 26 to 16 (Fig. 5).

### Case 7

A 46 year old male was diagnosed with AML del11q23 in 1996. He received chemotherapy followed by autoSCT. In 2012 he showed a progressive leukocytosis and AML with del 7 was diagnosed. He received induction chemotherapy with daunorubicin and cytarabine and obtained a 2nd complete remission. He received a PBSCT from an 8/8 HLA identical unrelated donor after myeloablative conditioning. He developed progressive scleroderma, non infectious hepatitis and mild diarrhea 2 years after transplantation with 18 kg of weight loss. Intense xerophthalmia, xerostomia and skin involvement was observed. He had alopecia, disabling acral erythema and desquamation, following development of lichen sclerosus-like lesions upon his trunk, arms, legs and ears. He developed severe sclerodactyly, resorption of distal phalanges and anonychia, with important functional impairment. Endoscopic study and biopsy confirmed cGVHD NIH grade 3. He was started on corticosteroids, tacrolimus, thalidomide, azathioprine, clofazimine without any response. Between December 2013 and October 2014, he was treated with ECP, with a transient intestinal and cutaneous response and a subsequent flare of cGVHD manifestations. A short cycle of methotrexate was inefficient; afterwards imatinib therapy during 6 months was also used without any improvement (A summarized information of all patients is showed in Table 1).

**Table 1:** Patients characteristics

Case	Gender/age	NIH GVHD grade	Sites involved	Previous treatment	Time to diagnosis to ruxolitinib	Response obtained	Follow up (years)	Dose adjustments of ruxolitinib due to adverse events
1	Male/51	3 acute	Skin Lung	Tacrolimus Mycophenolate Corticosteroids Extracorporeal photopheresis	21 days	Partial remission	2	No
2	Male/28	3 acute	Duodenal	Tacrolimus Mycophenolate Corticosteroids Extracorporeal photopheresis	5 weeks	Complete remission	2	No
3	Male/56	3 acute	Skin	Cyclosporine Methotrexate Corticosteroids Extracorporeal photopheresis	5 weeks	Complete remission	1	No
4	Female/26	3 chronic	Skin Oral mucosa Lung	Tacrolimus Mycophenolate Corticosteroids Extracorporeal photopheresis	3 months	Partial remission	1	Yes 50% reduction for neutropenia grade 2
5	Male/52	3 chronic	Skin Liver Oral mucosa	Tacrolimus Mycophenolate Corticosteroids Extracorporeal photopheresis	3 months	Partial remission	1	No
6	Female/36	3 chronic	Skin	Cyclosporine Methotrexate Corticosteroids Extracorporeal photopheresis	10 months	Partial remission	1	No
7	Male/46	3 chronic	Skin Liver	Cyclosporine Methotrexate Corticosteroids Imatinib Extracorporeal photopheresis	18 months	Partial remission	1	No
8	Female/26	2 chronic	Lung	Tacrolimus Mycophenolate Corticosteroids Extracorporeal photopheresis	6 months	Complete remission	1	Yes 75% reduction for neutropenia grade 2

**Fig. 6:**
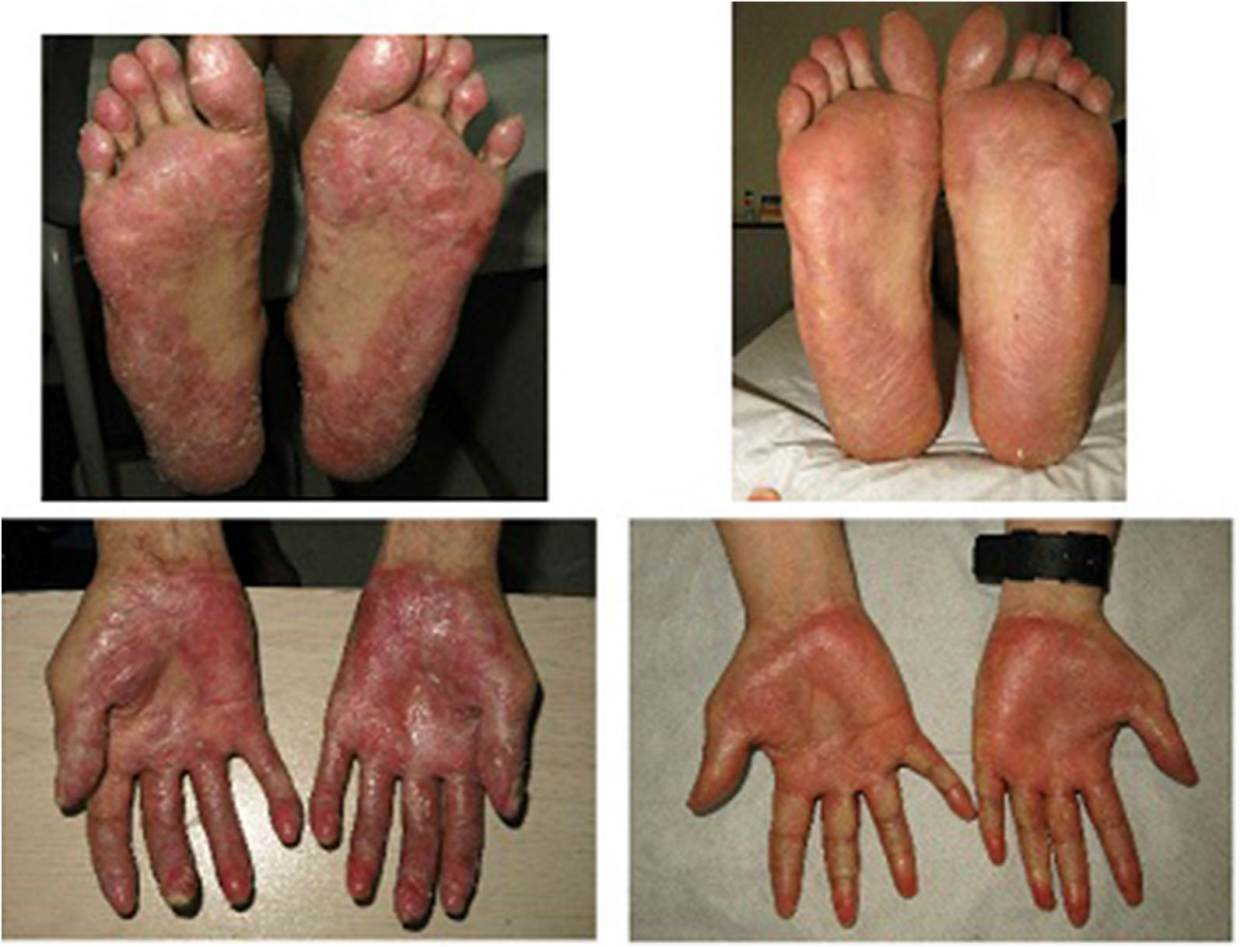
Left panels show intense sclerotic features of cGVHD in both hands and feet. Right panel shows a nearly complete resolution after ruxolitinib treatment

He started ruxolitinib 10 mg BID, and after 3 months hepatitis features resolved and diarrhea stopped. After 6 months of treatment he shows a significant improvement of scleroderma, he recovered his basal weight and shows a complete improvement of ocular GVHD (Fig. 6).

### Case 8

**Fig. 7:**
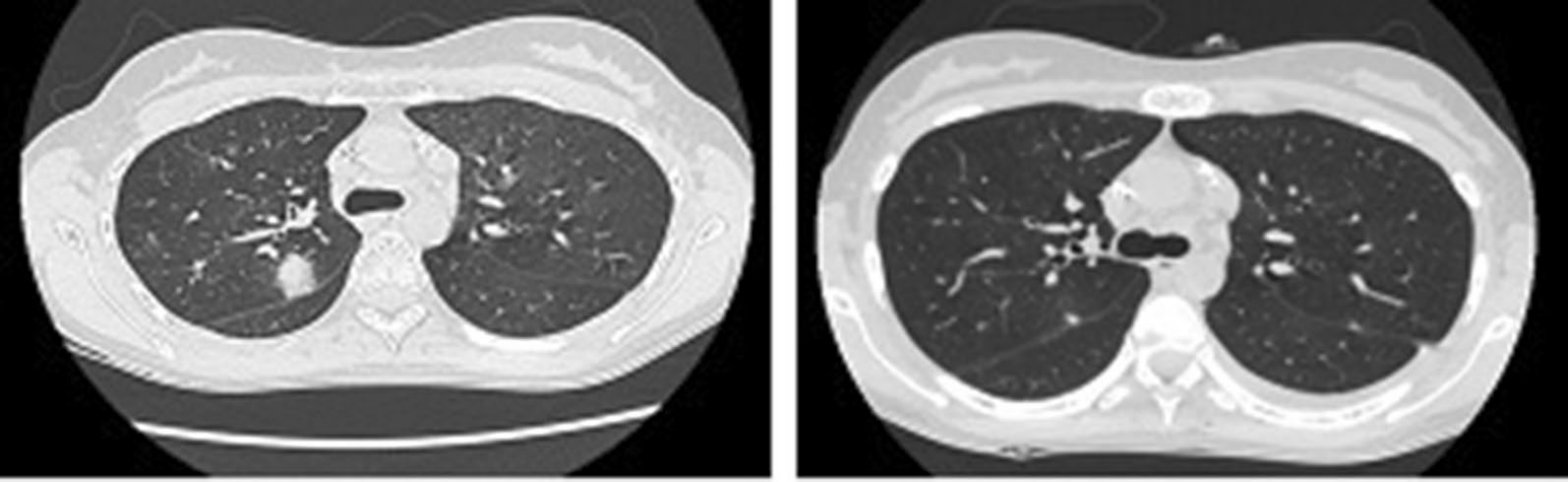
Left Pannel shows CT Scan at diagnosis of chronic lung GVHD. Right panel shows CT scan 3 months after ruxolitinb treatment

A 28 years old female was diagnosed with IV B classical Hodgkin lymphoma in 2014, and was treated with ABVD schema obtaining a complete remission. She relapsed in 2016 and after a first attempt of ICE chemotherapy no response was observed and brentuximab rescue was planned. She completed 3 months of treatment and attained a 2nd complete response. She received a PBSCT from a haploidentical donor after reduced intensity conditioning. 6 months post transplantation she developed a progressive hypoxemic pulmonary insufficiency and CT scan shows a nodular pattern. Bronchioloalveolar lavage was performed which ruled out any infection. A lung biopsy showed an inflammatory pattern compatible with GVHD NIH grade 3. There was no response to corticosteroid therapy and ECP was started without significant improvement after 12 sessions. Ruxolitinib 10 mg BID was started and after 3 months of therapy his CT Scan showed a complete remission of lung nodular infiltrates (Fig. 7).

## Discussion

GVHD is a potentially lethal complication after hematopoietic stem cell transplantation [3]. The absence of response to corticosteroids is associated with an increased risk of death. EP has allowed treating patients who do not respond to corticosteroids, with the advantage of being less immunosuppressive as compared to other strategies such as ATG, imatinib, pentostatin, alemtuzumab, rituximab, etc. However, patients who do not respond to corticosteroids and EP are in a very unfavorable situation. Usually these patients have been treated with different immunosuppressive treatments without a consistent response.

Ruxolitinib has emerged in recent years as an excellent strategy for the control of refractory patients [9]. Previously, preclinical studies showed that ruxolitinib have a potent immunomodulatory and anti-inflammatory effects with decrease of proinflammatory cytokines [10]. More recently, a cooperative study [11] showed a multicentric analysis of 54 patients with aGVHD refractory to corticosteroid treatment and 41 with cGVHD with overall response rate of 81.5% (44/54) in aGVHD including 25 complete responses (46.3%) and an ORR of 85.4% (35/41) in cGVHD, with a low GVHD relapse rate. This outstanding outcomes have also been reported in treatment of sclerotic forms of the disease, a condition that usually has poor response rates [12, 13]. Not only in adult transplantation has been reported this remarkable response. Recently a pediatric group showed a 45% overall response in 13 children’s with refractory GVHD [14]. Based on these encouraging results prospective randomized trials are ongoing in order to confirm the efficacy of ruxolitinib in acute and cGVHD. Also, a new JAK2/FLT3 inhibitor, pacritinib, has recently shown promising results in xenograft mouse models of JAK2V617F-driven diseases [15]. Likewise, there is a growing interest in developing other JAK pathway inhibitors in GVHD, such as momelotinib, bacritinib and itacitinib [16, 17]. To the best of our knowledge, our report is the first to show experience in the use of ruxolitinib in patients refractory or resistant to corticosteroids and EP. In our patients who have received this agent within a compassionate use, an overall response rate of 90% was observed. Another interesting report from our series is that, even in patients with digestive GVHD responses have been observed, despite the doubts regarding the bioavailability of oral drugs in patients with high-flow diarrhea [18]. In addition, we observed a favorable response in pulmonary GVHD, the most severe and with the highest mortality [19]. So far all of our patients continue to use the drug, with good tolerance and without major adverse effects. Only in two of them was it necessary to adjust the dosage due to haematological toxicity, mainly mild neutropenia, which was recovered when establishing the appropriate dose. Our report shows a satisfactory experience in controlling this serious condition among patients failing to corticosteroids and EP. According to our experience, it could be thought that it is better to use ruxolitinib as a second-line medication for the treatment of refractory GVHD. Randomized studies are necessary to answer this and generate a treatment algorithm.

## Conclusions

Ruxolitinib has shown a remarkable and hopeful response in patients with GVHD refractory to corticosteroids in recent years. Our report shows that in patients with refractoriness to corticoids and extracorporeal photopheresis also has excellent results with good tolerance and minimal adverse effects, even in difficult cases such as sclerodermiform forms and lung disease.

## Abbreviations

AML: acute myeloid leukemia
ABVD: chemotherapy protocol with doxorrubicina, bleomycin, vinblastine, dacarbazine
CR: complete remission
CMV: Cytomegalovirus
EBV: Epstein Barr virus
EP: extracorporeal photoapheresis
FLAGIDA: chemotherapy protocol with fludarabina, idarrubicin, cytarabine, filgrastim
GVHD: graft versus host disease
HyperCVAD: chemotherapy protocol with cytarabine, vincristine, doxorrubicin, dexamethasone, methotrexate
ICE: chemotherapy with ifosfamide, carboplatine, etoposide
IL: interleukin
IFN: interferon
JAK2: janus kinase
PBSCT: peripheral blood hematopoietic stem cell transplantation
PET/CT: positron emission tomography
